# Tubulin Isotypes: Emerging Roles in Defining Cancer Stem Cell Niche

**DOI:** 10.3389/fimmu.2022.876278

**Published:** 2022-05-26

**Authors:** Tessy Thomas Maliekal, Dhrishya Dharmapal, Suparna Sengupta

**Affiliations:** ^1^Cancer Research, Rajiv Gandhi Centre for Biotechnology, Thiruvananthapuram, India; ^2^Regional Centre for Biotechnology, Faridabad, India; ^3^University of Kerala, Department of Biotechnology, Thiruvananthapuram, India

**Keywords:** tubulin, tubulin-interacting proteins, cancer stem cell niche, metabolic reprogramming, immune evasion, GLUT1, GRP78, EphrinB1

## Abstract

Although the role of microtubule dynamics in cancer progression is well-established, the roles of tubulin isotypes, their cargos and their specific function in the induction and sustenance of cancer stem cells (CSCs) were poorly explored. But emerging reports urge to focus on the transport function of tubulin isotypes in defining orchestrated expression of functionally critical molecules in establishing a stem cell niche, which is the key for CSC regulation. In this review, we summarize the role of specific tubulin isotypes in the transport of functional molecules that regulate metabolic reprogramming, which leads to the induction of CSCs and immune evasion. Recently, the surface expression of GLUT1 and GRP78 as well as voltage-dependent anion channel (VDAC) permeability, regulated by specific isotypes of β-tubulins have been shown to impart CSC properties to cancer cells, by implementing a metabolic reprogramming. Moreover, βIVb tubulin is shown to be critical in modulating EphrinB1signaling to sustain CSCs in oral carcinoma. These tubulin-interacting molecules, Ephrins, GLUT1 and GRP78, are also important regulators of immune evasion, by evoking PD-L1 mediated T-cell suppression. Thus, the recent advances in the field implicate that tubulins play a role in the controlled transport of molecules involved in CSC niche. The indication of tubulin isotypes in the regulation of CSCs offers a strategy to specifically target those tubulin isotypes to eliminate CSCs, rather than the general inhibition of microtubules, which usually leads to therapy resistance.

## Introduction

Microtubules, a major class of the cytoskeleton of cells, are formed of heterodimers of α and β tubulins (1). In addition to the heterogeneity of the tubulin isotypes forming the dimers, their post-translational modifications and interacting proteins influence the dynamics of microtubules (2, 3). As microtubules regulate plethora of cellular processes, their deregulation is associated with diseases, including cancer. As the field evolves, it appears that the tubulin isotypes might have unique functions specified by their interacting molecules, which again is defined by their unique C-terminal end sequence and the post translational modifications (PTMs) therein (2). Deregulation of some of the tubulin isotypes in cancer suggests their involvement in cancer progression. Though microtubule targeting agents (MTA) have been used in cancer treatment for a long time, the role of the tubulin isotypes in the cancer stem cell (CSC) context was not explored extensively. Some of the recent findings suggest that specific tubulin isotypes have some important roles in regulating certain functionally essential molecules involved in the induction and maintenance of CSCs (4–8). This review focuses on the molecules that are shown to interact with tubulin isotypes, probably their cargos. We discuss the recent reports portraying the role of tubulin isotypes and their plausible cargos in metabolic reprogramming, induction of CSCs and modulation of immune evasion

## The Tubulin Code

**Figure 1:**
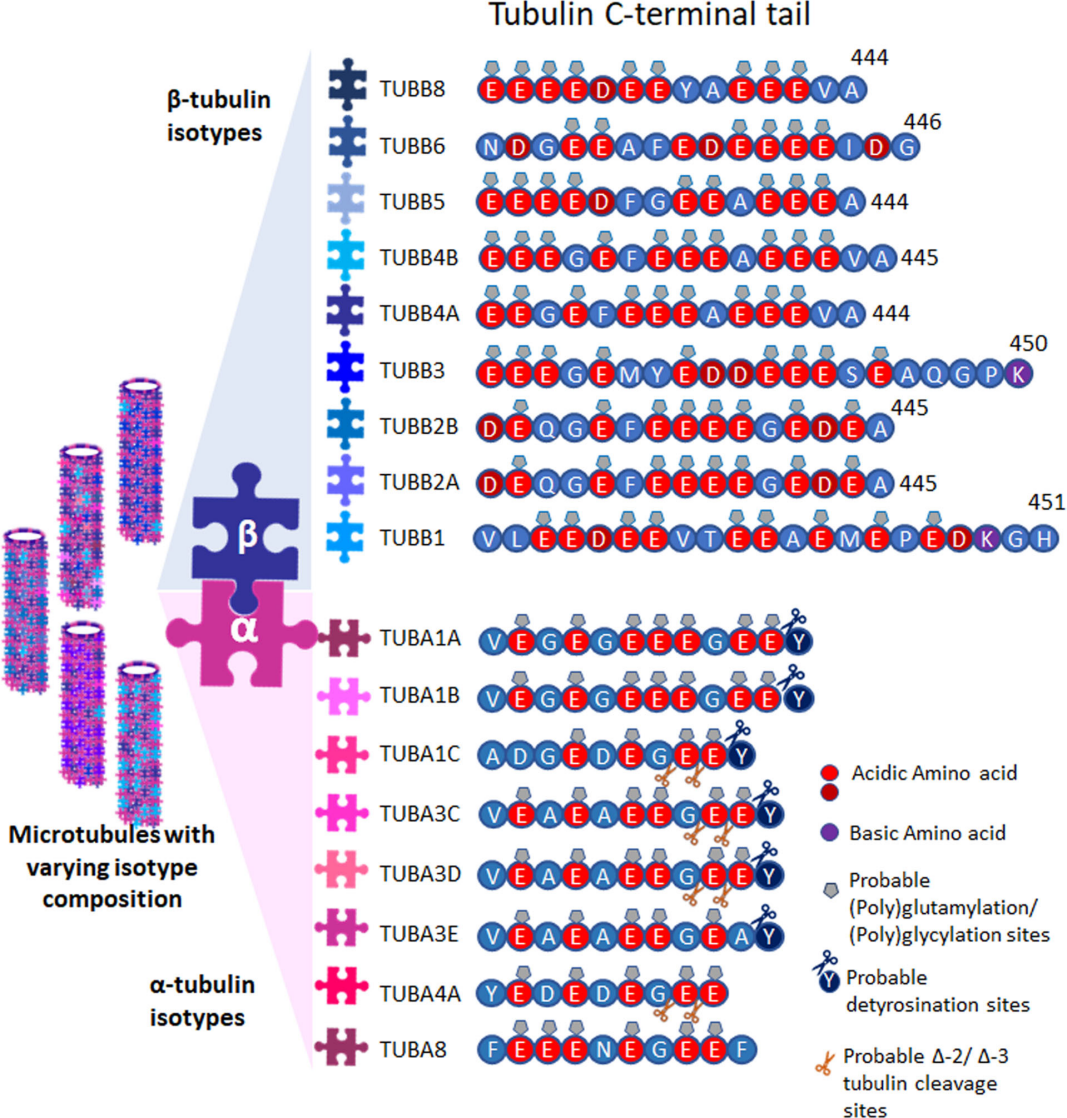
Tubulins and their PTMs. Tubulin isotypes depicting the varying C-terminal sequences, showing probable post translational modification sites. Microtubules are formed with varying isotype composition.

Several isotypes of tubulin are found for both α- and β-tubulins in different species. So far, mammals are known to have nine α- tubulin and nine β-tubulin genes, (**Figure 1**) (for nomenclature see: www.genenames.org/cgi-bin/genefamilies/set/778). The isotype composition, differing majorly in the 20 amino acids of their extremely acidic C-terminals, plays a critical role in imparting structural conformation, motor activity and tunes the microtubule dynamics (1, 3), which turns out to be the reason for the unique functions of particular tubulin isotype combinations. The concept that molecular patterns generated by combinations of tubulin isotypes and PTMs is termed the ‘tubulin code’. The tubulin molecule with highly conserved core can have slightly different structures with different isotype incorporation affecting microtubule assembly, dynamics and mechanical properties. Also, the isotypes with unique flexible tails that protrude outward from the surface of microtubules affect the interaction with microtubule associating proteins (MAPs) and thus can cause unique PTMs. Further, this variation is multiplied by a plethora of PTMs such as acetylation, tyrosination, detyrosination, glutamylation, polyglutamylation, glycation, phosphorylation etc (2). Isotype overexpression or down-regulation can bring in several diseases including neuronal disorders (9), cancer (10), drug resistance (11, 12) etc. So far it was not possible to assign the sequence difference of specific isotypes to different functions but it can be assessed that the variable stretch of negative charges over the C-terminus along with PTMs incorporated might play a significant role in defining the unique functions.

## Microtubule-Mediated Transport

**Figure 2:**
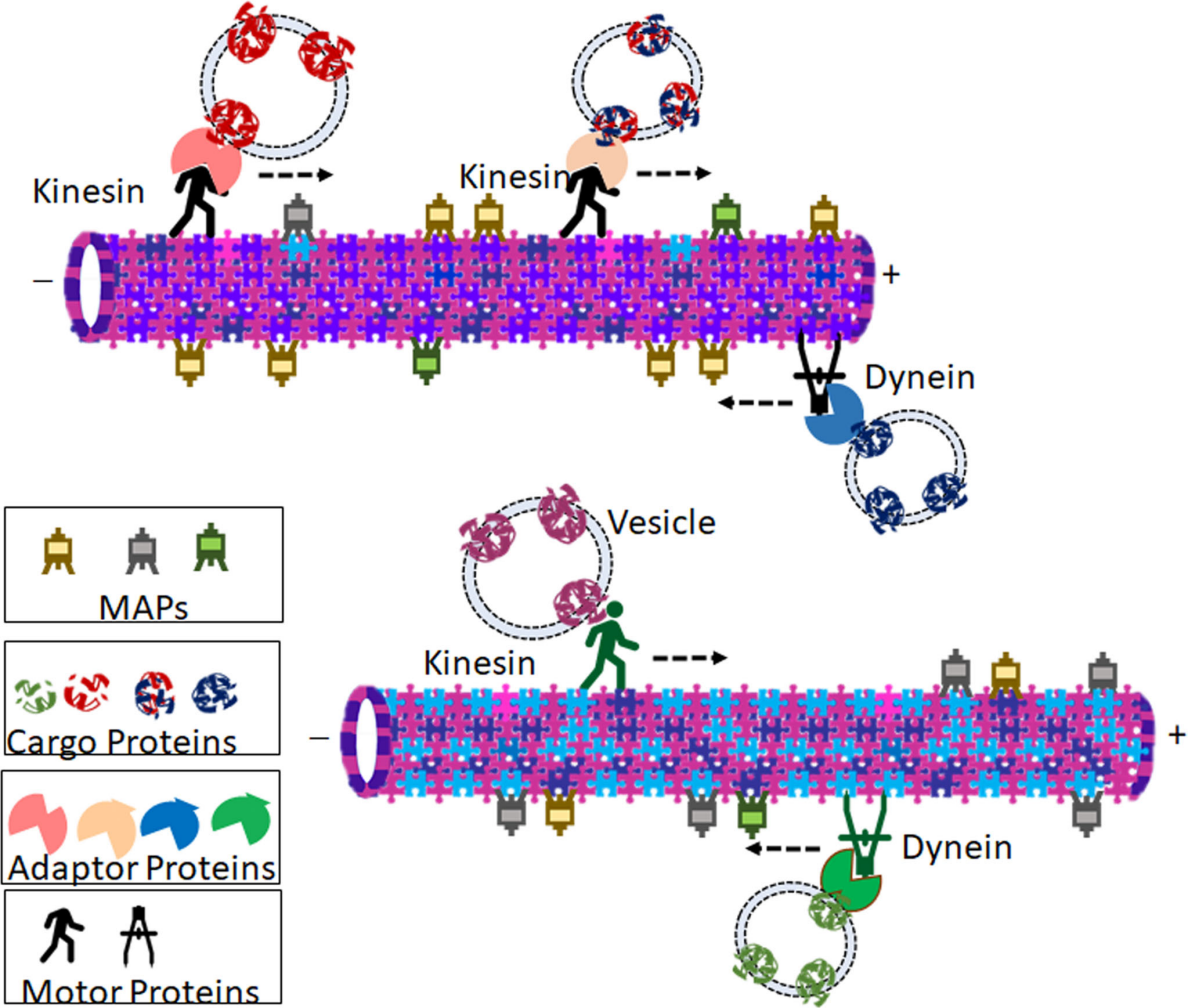
Representation of probable theory that microtubule isotype composition contributes to specificity in tubulin-mediated transport. Cargos (Vesicle bound proteins or Organelles) are transported in retrograde and anterograde directions by motor proteins Dyneins and Kinesins respectively. Many of the motor proteins bind to the vesicles through adaptor proteins. Isotype composition of the microtubule influences preferential binding of microtubule-associated proteins (MAPs), motor proteins, and ultimately the cargo is transported. The cartoon shows two representative microtubules with different isotype compositions. The varied relative abundance of different isotypes imparts the microtubules with diverse affinities towards different kinesins, dyneins, as well as MAPs. Due to the difference in isotype composition, the two microtubules carry different cargoes owing to the different motor proteins and adaptor proteins that bind to them. The association of different tubulins with different MAPs imparts varied microtubule kinetics.

Microtubules act as tracks for long-range intracellular transport of many important vesicles and organelles through its motor proteins kinesins and dyneins (**Figure 2**). They are critical in the positioning of golgi complex (13), endoplasmic reticulum motility (14), long-distance transport of mitochondria (15) as well as the translocation and clustering of endosomes and lysosomes (16, 17). Microtubules mediated trafficking has negative effects also. For example, pathogenic viral cargos require microtubules to transport them to and from their intracellular replication sites. The microtubule-mediated transport of cargo with spatiotemporal specificity and efficiency demands the involvement of a set of other proteins. Some of the MAPs, identified initially as the proteins that bind to and stabilize microtubules, specifically Tau, MAP1B, MAP2, MAP4, MAP6, and MAP7, are now considered to be the regulators of intracellular traffic (18). The MAPs, specifically the plus-end-tracking proteins (+TIPs) that respond to various cellular signals, regulate the dynamic behavior and organization of the microtubule tracks (19). While the motor proteins power the transport, modifications of adapter proteins-the molecules that recruit cargos to the motor- fine tunes the specificity of transport (20). At the same time, some cargos can directly interact with their motors (21). Septins are multimeric GTPases that function as adapter proteins, which brings about the selective recruitment of microtubule motors to their respective cargo (22). Another such adapter molecule is c-jun NH_2_-terminal kinase (JNK)–interacting proteins (JIPs), which are scaffolding proteins for the JNK signaling pathway (23). JIP1, the kinesin-1 cargo, is localized only to a subset of neurites in cultured neuronal cells. This polarized protein trafficking appears to involve the preferential recognition of kinesin-1 motor domain to microtubules containing specific posttranslational modifications (PTMs) such as α-tubulin acetylation at Lys-40 (24). However, this PTM alone is not sufficient to affect kinesin-1 velocity and run length (25). It is becoming increasingly evident that the tubulin code with different isotype composition and posttranslational modifications plays an extremely important role in controlling motor behaviors and the dynamicity of microtubules (1, 26). Yet, despite decades of extensive research, our knowledge on the spatiotemporal regulation of microtubule is incomplete (27). The mechanism of cargo specificity during microtubule transport is still a mystery, as there are fewer known adapters than the number of cargos (27). In this context, some of the recent reports of the direct interaction of some functional proteins, like VDAC, N-Cadherin, GLUT1 and EphrinB1, to specific tubulin isotypes, suggests additional mechanism of implementing specificity of transport (4–6, 28).

## Tubulins in Cancer

Consistent with the divergent role of tubulins in cell cycle regulation, apoptosis and drug resistance, their deregulation has been reported in a wide range of cancers. Aberrant expression of certain isotypes of tubulins in cancer tissue specimens are reported to regulate cancer progression, metastasis, aggressive behavior, drug resistance or poor prognosis, as summarized in **Table 1**. The upstream signaling leading to this aberrant expression is not well-characterized except for βIII-tubulin (44). As summarized in a recent review, several factors including hormones, bromodomain and extraterminal (BET) proteins, factors like hypoxia and hypoglycemia can up-regulate the expression of βIII-tubulin (44). While several upstream signaling pathways like AKT, K-RAS and EGFR upregulate its expression, tumor suppressor PTEN negatively regulates the gene expression (44). The upstream pathways that regulate the PTMs of the rest of the tubulin isotypes are yet to be unraveled.

**Table 1:** Tubulin deregulation in cancer.

Isotype	Alteration	Cancer	Outcome	Reference
α-tubulin*	Loss of expression	Breast cancer	Metastasis	(29)
	Increased acetylation	Breast cancer	Metastasis, Aggressive behavior	(30)
αIa-tubulin, αIb-tubulin	Over-expression	Breast cancer	Taxane resistance	(31)
βI-tubulin	Over-expression	Breast cancer	Docetaxel resistance	(32)
	Over-expression	Breast cancer	Taxane resistance	(31)
βII-tubulin*	Increased mRNA	Nasopharyngeal carcinoma	Cancer progression	(33)
	Over-expression	Colorectal cancer	Poor outcome	(34)
	Nuclear localization	Variety of cancers	Metastasis	(35)
βIIa-tubulin	Expression	Breast cancer	Metastasis	(36)
βIIb-tubulin	mRNA expression	Colorectal cancer	Poor survival	(37)
	mRNA expression	Renal cancer	Poor survival	(38)
	Down-regulation	Breast cancer	Taxane resistance	(31)
βIII-tubulin	Over-expression	Breast cancer	Docetaxel resistance	(32)
	Over-expression	Breast cancer	Taxane resistance	(31)
	Over-expression	Clear cell renal carcinoma	Poor prognosis	(39)
	Over-expression	Prostate cancer	Docetaxel resistance	(40)
	Over-expression	Colorectal cancer	Poor prognosis	(41)
	Over-expression	HNSCC	Unrelated to clinical outcome	(42)
βIVb-tubulin	High mRNA Expression	HNSCC	Unrelated to clinical outcome	(5)
βV-tubulin	Over-expression	NSCLC	Prolonged progression-free survival	(43)
	Down-regulation	Breast cancer	Taxane resistance	(31)

Reported deregulation of Tubulins in tissue samples of different cancers. Data from cell lines are not included. HNSCC, Head and neck squamous cell carcinoma; NSCLC, non-small cell lung carcinoma. *References where isotypes are not mentioned.

The role of different isotypes of tubulins in cancer progression is reviewed recently (45, 46). It is well-established that microtubule network and their dynamics are important regulators of mitosis and cell proliferation (45). Since microtubule dynamics also plays a role in cell migration, tubulins, more specifically their post-translational modifications, regulate invasion and metastasis (34, 45, 47). Further, tubulins also play a critical role in the regulation of drug resistance (45). The most studied post-translational modification of tubulins that regulate different cancer properties is acetylation. Acetylation of different isotypes of tubulin is shown to regulate the invasive property, metastatic ability and resistance to chemotherapy (30, 48). The role of different posttranslational modifications, including acetylation, detyrosination, tyrosination, polyglutamylation, and polyglycylation in the regulation of cancer properties are extensively reviewed elsewhere (49).

The classical research on the role of tubulins in cancer revolves around the microtubule dynamics that change the biophysical properties of the cancer cell. But recent evidences implicate that tubulins can indirectly regulate many cancer properties including drug resistance, metastasis and immune evasion by the transport of important molecules necessary for the maintenance of cancer stem cells (CSCs) and their niche (4–8). Majority of the studies in the field were focused on the tubulin-interacting molecules that regulate the dynamics of microtubules like motor proteins and MAPs (50). Yet, there are some studies that showed that molecules other than microtubule associated proteins and motor proteins co-immunoprecipitates with specific tubulins, suggesting the specificity of isotypes for selecting the cargo (**Table 2**). So far, there is only one study reported using proteomic approach to identify the interacting partners of βIII-tubulin, which revealed that the molecule forms complexes with important regulators like GRP78, Vimentin and GSTM4 in cancer cells (56). Interestingly, nuclear βII-tubulin is shown to associate with Notch1 intracellular domain (55). Recently, it is also shown that S100A6 binds to alpha and beta tubulins, and the secretion of S100A6 is dependent on its tubulin-binding (7). Another important observation linking tubulins to mitochondrial bioenergetics function is the regulation of voltage-dependent anion channel (VDAC) permeability by βII-tubulin and βIII-tubulin (4, 57). Also, it was found that βIVb-tubulin interacts with GLUT1 in the CSC context (6). Moreover, we recently reported the involvement of βIVb-tubulin in the possible transport of EphrinB1 in the CSC niche (5). **Table 2** gives a summary of the known interacting molecules of tubulins, which might have a role in the regulation of cancer properties. Though the role of respective tubulins in their transport is not well-established in certain cases, it opens up the possibility of tubulins playing a role in specific transport of important intermediates of proliferation, apoptosis, chemoresistance, CSC properties and immune evasion. In the following sections, we will elaborate on how the transport function of tubulins can possibly regulate the cancer properties.

**Table 2:** Tubulin interacting proteins.

Tubulin type	Interacting Molecule	Interaction context	Reference	Relevance of interacting molecule in cancer
α-tubulin	Vimentin	Colon cancer migration	(51)	Metastasis
	VDAC1	Lung cancer cells	(52)	Metabolic reprogramming in cancer
α-tubulin and βI-tubulin	RAMP1	Mouse TSA cells and Human SH-SY5Y neuroblastoma cells	(53)	Increase proliferation
α-tubulin and β-tubulin	EAG2	Human brain medley	(54)	Increase proliferation and cancer progression
α-tubulin and β-tubulin	S100A6	Regulates secretion of S100A6 in mesenchymal stem cells, WJMS	(7)	Metastasis
αIa-tubulin, and βIVb-tubulin	Connexin43	Mouse brain	(8)	Increase proliferation, metastasis, inhibit apoptosis
β-tubulin	Connexin43	HeLa cells	(8)	Increase proliferation, metastasis, inhibit apoptosis
βII-tubulin	Notch1-NIC	Nuclear translocation of Notch in Leukemia cells	(55)	Regulate CSCs
βIII-tubulin	Vimentin	Ovarian cancer cells	(56)	Metastasis
	GRP78	Ovarian cancer cells	(56)	Regulate CSCs
	GRP75	Ovarian cancer cells	(56)	Regulate proliferation, survival and CSC properties
	GSTM4	Ovarian cancer cells	(56)	Drug resistance
βIII-tubulin and βIVa-tubulin	N-Cadherin	Endothelial cells	(28)	Metastasis
βIVb-tubulin	GLUT1	Glioblastoma stem cell niche	(6)	Regulate CSCs
	EphrinB1	Oral cancer stem cell niche	(5)	Regulate CSCs

The table enlists molecules that are reported to interact with tubulins, confirmed by immunoprecipitation.

## Tubulins and Their Interacting Proteins in the Regulation of Cancer Progression

Given the role of tubulins in the regulation of mitosis, a number of chemically diverse substances are developed that bind to tubulin and inhibit cell proliferation by disrupting the microtubule dynamics, activating spindle assembly checkpoints and mitotic arrest (58). The recent advances in the field suggest that tubulin isotypes other than γ-tubulins might have a microtubule-mediated spindle assembly-independent role in proliferation (54). Although the voltage-gated potassium channel EAG2 is shown to interact with α-tubulin and β-tubulin, the possible role of this interaction in its trafficking and function in tumor progression is yet to be studied (54). However, EAG2 plays a role in cancer progression, as shown in medulloblastoma (59). It is shown to be up-regulated in medulloblastoma tissues and its knock-down impairs medulloblastoma cell growth *in vitro*, reduces tumor burden *in vivo* and enhances survival in xenograft studies (59). Connexin43 is a gap junction protein shown to be associated with tubulins, and it is involved in cancer progression by modulating MAPK and JNK pathways (60–63). One of the other properties attributed to tubulins is cell migration. Hence, it is implicated in metastasis and tubulin inhibitors are shown to block metastasis in various cancers (47, 64, 65). Now there are accumulating evidences to show that tubulins can regulate cancer properties other than proliferation and metastasis. Some of the recent studies show that certain isotypes of tubulins are associated with CSCs, and the knock-down of those isotypes can deplete CSCs (5, 6, 39).

## Tubulins Implicated in the Regulation of CSCs and Their Niche

**Figure 3:**
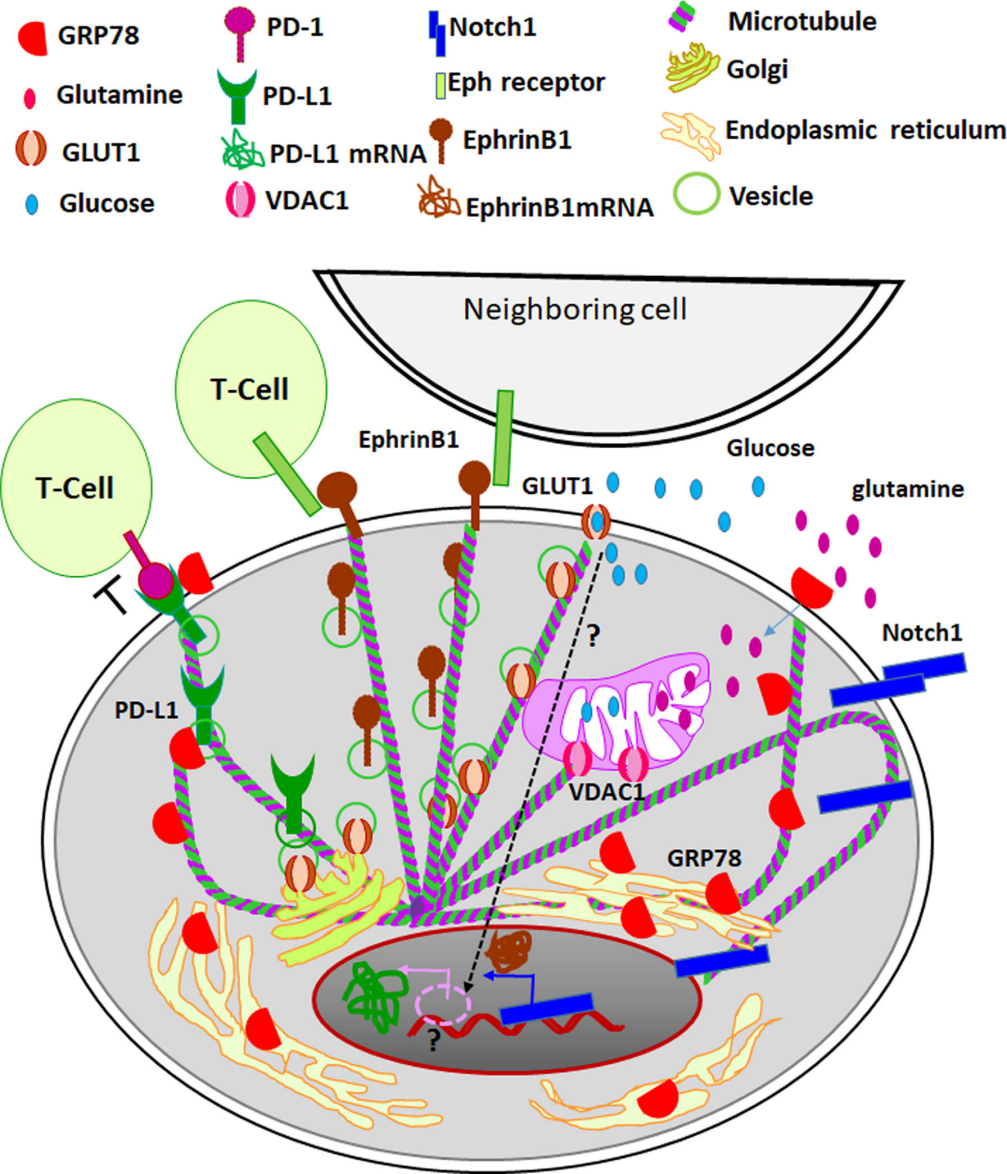
The role of tubulins in CSC niche. Different tubulin isotypes regulate metabolic reprogramming important for the induction of CSCs. While hypoxia is a critical factor that activates the expression of GLUT1-the regulator of aerobic glycolysis and metabolic reprogramming, its surface localization is controlled by βIVb tubulin. Also, other isotypes of tubulin, βII and βIII, regulate the opening and closing of VDACs, which also is a crucial regulation of metabolic reprogramming. The endoplasmic reticulum residing GRP78 is shown to surface localize during cellular stress, the transport of the molecules is thought to be modulated by βIII tubulin. The surface localized GRP78 enhances glutamine intake and its metabolism, which also contributes to the metabolic reprogramming. In parallel, certain master regulators of self-renewal, like Notch1 leads to transcriptomic regulation for the induction of stemness. The nuclear transport of the active cleaved Notch1 is facilitated by βII tubulin. EphrinB1, a target gene of Notch 1, leads to Eph/Ephrin bidirectional signaling. The surface transport of EphrinB1 is shown to be dependent on βIVb tubulin. Eph/Ephrin signaling regulates the fate of cancer cells and immune infiltrates. When PD1 on T-cells is engaged by its ligand PD-L1, it leads to the inhibition of T-cell activity. The aberrant expression of PD-L1 on cancer cells leads to immune evasion. While the expression of PD-L1 is indirectly regulated by GLUT1 activity through unknown mechanism (shown as dotted arrow and question mark), stabilization of its surface localization is shown to be mediated by GRP78. Thus different specific isotypes of tubulins are involved in the transport of critical molecules involved in metabolic reprogramming, induction of stemness and immune evasion in CSC niche.

As summarized in **Table 1**, specific isotypes of tubulins are enriched in certain cancers, which lead to the acquisition of resistance to tubulin-binding agents. Thus, several studies have investigated the role of different isotypes in imparting resistance, which revealed that the dynamics of βIII tubulin comprising microtubule are quite different from the ones composed of mixed β tubulins (10, 46). Further, binding efficiencies of different microtubule-targeting agents (MTA) to microtubules comprising βIII tubulins are lower compared to their efficiencies to bind to microtubules consisting of different β tubulins (10, 46). Though the expression profiles of tubulin isotypes in cancer and chemoresistance are well-explored, the significance of tubulin isotypes in the regulation of CSCs is poorly studied. However, a recent study using several glioblastoma cell lines has shown that there was a reduction in the detyrosinated form of α-tubulin, acetylated α-tubulin and phosphorylated βIII-tubulin with a concomitant up-regulation of polyglutamylated α and β-tubulins in MTA-resistant cells compared to sensitive cells. Also, the MTA-tolerant cells expressed stemness markers, suggesting that CSCs exhibit resistance to MTA (66). The MTA-resistant glioblastoma cell lines had a relative enrichment of βII and βIII tubulins, while the detyrosination and the change in Δ-2 α-tubulin levels were not correlated to resistance (66). Shortly after that, a more direct evidence of the role of βIII tubulins in clear cell renal cell carcinoma stem cells was reported (39). They showed that there is a positive correlation between the expression of βIII tubulin and stem cell markers (39). In accordance with that, the depletion of βIII tubulin resulted in the loss of CSC properties (39). Recently, our studies in oral cancer have shown that βIVb-tubulin is indispensable for the maintenance of CSCs, specifically in defining the CSC niche (5). Further, it was found to be critical for the maintenance of glioblastoma stem cells (6). A detailed analysis of the recent literature suggest that different tubulin isotypes are involved in the regulation of CSCs and their niche, possibly by the transport of essential molecules that regulate CSC properties. The important CSC-regulating molecules that are suggested to be transported by tubulins are signaling molecules like, Ephrins and Notch; regulators of metabolism like, GLUT1, GRP78 and VDAC (**Table 2**). Many of these molecules present in CSC niche are important in establishing metabolic reprogramming, imparting self-renewal ability and to some extent, to facilitate immune evasion (**Figure 3**).

### Metabolic Reprogramming in CSC Niche

In order to meet the requirements of exponential growth and proliferation, cancer cells adapt to aerobic glycolysis, glutamine catabolism, *de novo* lipid synthesis and nucleotide synthesis, which is generally known as metabolic reprogramming (67). The switch of tumor cellular bioenergetics from an oxidative phosphorylation to aerobic glycolytic pathway is now recognized as one of the hallmarks of cancer (68). At the same time, it is also shown that CSCs have metabolic plasticity, and can switch between mitochondrial respiration and glycolysis (69) Research on this topic for the last two decades has shown that metabolic reprogramming is the key to the induction of CSCs (70, 71). The most convincing link between tubulins, cellular bioenergetics and CSCs is the role of tubulin-VDAC axis in the metabolic reprogramming in cancer cells (72). VDACs are located in the mitochondrial outer membrane, which function as a metabolic link between glycolysis and oxidative phosphorylation (72). Both βII and βIII tubulins are found to regulate VDAC channel permeability in normal and cancer cells (57). The dynamic regulation of free and dimerized tubulins regulate VDAC opening and closing to modulate mitochondrial metabolism, reactive oxygen species formation, and the intracellular flow of energy (72). In accordance with that, cytoskeleton-mitochondrial interactions through VDACs are implicated in the regulation of CSCs (73).

One of the first reports that link microtubules to glucose metabolism is the observation that D-glucose induces tyrosination of tubulins (74). The tyrosination of tubulins, in turn, has been shown to regulate the motor protein-mediated transport in the nervous system (75, 76). Following up the research on the transport function of tubulins unraveled the role of tubulins in the transport of many molecules involved in the regulation of glucose metabolism in CSCs and their niche. Hypoxia, the most important factor leading to the metabolic reprogramming and induction of CSCs, is shown to induce GLUT1, the transporter of glucose into cells (77, 78). The metabolic reprogramming in CSCs is shown to be dependent on GLUT1 in many cancer types (6, 79–81). Remarkably, GLUT1 is shown to interact with βIVb-tubulin in glioblastoma specimens using mass spectrometric analysis, which was confirmed again by proximity ligation assay and immunoprecipitation (6). Of note, this depletion of the tubulin reduced the surface expression of GLUT1, and resulted in the loss of CSC properties (6), thereby highlighting the role of βIVb-tubulin in the membrane transport of GLUT1 and the regulation of stem cells thereby. Like GLUT1, another important regulator of metabolic reprogramming, GRP78, is shown to associate with tubulins. It is shown that βIII tubulin interacts with GRP78, and this interaction is critical for the survival of cancer cells in the glucose starved condition by adapting to use other nutrient supplies present in the tumor microenvironment (82). This GRP78-mediated survival is shown to be dependent on the enhanced glutamine catabolism (83). Thus, GRP78, more specifically the cell surface GRP78, regulates metabolic reprogramming, which depends on several other molecular players (84, 85). Whether the reported interaction of tubulins to GRP78 plays any role in its cell surface function is yet to be studied.

### Regulation of Self-Renewal of CSCs

The over-expression of βII tubulin and its nuclear localization is reported as a marker for poor prognosis in colorectal cancer (34). The importance of this βII tubulin-nuclear localization in the aggressive nature of the malignancy was explained by another study that demonstrated the involvement of tubulin βII in the nuclear transport of Notch1 and its CBF-dependent transcriptional activity (55). Notch1 is supposed to be a master-regulator of stem cell properties, as it controls a variety of molecules that regulate CSCs and their niche in various malignancies (86–89). Notch signaling is important in the maintenance of stem cells in intestinal crypts, by regulating the expression of EphrinB1, where the reciprocal gradients of EphB2 and EphrinB1 define the balance of intestinal stem cell self-renewal and differentiation (90). In cancer context also Notch1 is reported to regulate EphrinB1 signaling, as shown in osteosarcoma (91). Notably, another tubulin isotype, βIVb is shown to directly regulate EphrinB1 surface localization in oral cancer stem cells and their niche (5). Also, abrogation of βIVb tubulin or EphrinB1, which reduced the surface expression of EphrinB1 or the active EphrinB1 signaling, depleted the CSC population (5). Thus different tubulin isotypes play specific roles in the maintenance of CSCs by transporting unique signaling molecules involved in the regulation of stemness.

### Modulation of Immune Evasion

In spite of the ability of immune cells to actively eliminate transformed cells, cancer cells survive in our body by manipulating the immune response machinery (92). This machinery, including CD4+ and CD8+ T cells, dendritic cells (DCs), and natural killer (NK) cells, is usually inhibited by certain checkpoint molecules, as a part of natural feedback inhibition, to prevent excessive immune reactivity (92). Cancer cells cleverly overexpress these checkpoint molecules including programmed death receptor ligands (PD-L1/PD-L2), and cytotoxic T cell-associated antigen-4 (CTLA-4) to evade the immune response (92). A growing body of evidence has demonstrated the active cross-talk of CSCs and immune infiltrates within the CSC niche (92). While the activity of some immune cells supports the expansion of CSCs, this subpopulation of cancer cells actively elicit immune evasion through a number of distinct mechanisms (92, 93). The metabolic reprogramming leading to the generation of CSCs can also regulate the immune evasion, as some of the metabolites produced by the alternative metabolism in the tumor microenvironment, like lactic acid and extracellular adenylate can regulate the fate of infiltrating immune cells (94). A more important aspect of metabolic reprogramming is the enhanced glycolytic activity-dependent up-regulation of PD-L1 that leads to the inhibition of cytotoxic T-cells (94). So tubulins as a mediator of metabolic reprogramming might regulate immune evasion also.

A recent study identifying immune related gene signatures in pancreatic cancer identified βIII tubulin as a critical immune regulator, closely linked to the T-cell receptor signaling pathway (95). This observation is in accordance with the earlier reports showing the importance of tubulin dynamics and molecular motors in immune synapse of T-cells and antigen presenting cells (96). Microtubule inhibitors, either anti-depolymerization agents such as the taxane family, or anti-polymerization agents such as colchicine and vinca alkaloids, have different effects on immune cell isotypes (97). Majority of the reports showing the effect of taxanes on immunemodulation attributes the activity of the drug on T-cells (97). Likewise, a widely used anti-polymerization agent, colchicine, down-regulates most immune cell types (97). Even though these reports showed the importance of tubulins and their cargos in T-cells for its function, there are some evidences to show that tubulins of the cancer cells, more specifically their inhibitors, can play a role in modulating immune response. One of the recent reports has shown that microtubule targeting agents, like vinca alkaloids and colchicine, can up-regulate the expression of PD-L1, a critical regulator of immune evasion (98). In clear cell renal cell carcinoma tissues the expression of βIII tubulin was associated with PD-L1 (39). Mechanistically, this correlation might be dependent on some of the tubulin interacting molecules. In agreement to the critical role of GLUT1 in glycolysis and metabolic reprogramming, the expression of PD-L1 is shown to depend on the activity of GLUT1 (99). Further, GRP78 is shown to physically interact with PD-L1 to increase its stability (100). Consistent with that, the enhanced expression of both PD-L1 and GRP78 is correlated with poor relapse-free survival in triple-negative breast cancer (100). As the different Ephrin ligands can engage the Eph receptors on immune cells and modulate their activity, Eph/Ephrin signaling is considered as a mediator of tumor immunity. More importantly, Eph/Ephrin signaling within the cancer cells can up-regulate the expression of PD-L1 (101). Since tubulins are shown to interact with these molecules, possibly regulating their localization and activity, specific tubulin isotypes present in the tumor microenvironment and/or CSC niche might be critical in the regulation of immune evasion.

## Conclusion

Given the importance of the niche in the regulation of properties of stem cells or cancer stem cells, the mechanism involved in the generation of a niche is of prime importance. When we merge recent studies in the CSC field with tubulin research, a hypothesis of tubulin-mediated transport in defining stem cell niches emerge. Tubulins might be playing a role in the orchestrated expression of ligands and receptors to facilitate active signaling. On a broader concept, this transport mechanism might be critical in several scenarios, where coordinated localization of functional molecules is required. As this aspect of tubulins are poorly explored, extensive research on this is warranted to understand the mechanism behind establishing niches. In the cancer context, understanding the central players in defining CSCs has immense therapeutic potential. If we identify the specific isotype of tubulins responsible for defining CSC niche, strategies can be developed to target only those isotypes instead of inhibiting the whole cytoskeleton, which generally leads to chemoresistance.

## Author Contributions

TM conceived the idea and prepared the manuscript. DD contributed in the preparation of figures. SS contributed to the preparation of manuscript and critically modified it. All authors contributed to the article and approved the submitted version.

## Funding

The work was supported by the institutional core funding from the Department of Biotechnology to SS and TM; and extramural grant to TM from Department of Science and Technology (SR/S0/HS/0133/2010) and Department of Biotechnology (BT/PR14379/Med/30/536/2010). DD is thankful to the University Grants Commission (22/06/2014(i)EU-V) and Indian Council of Medical Research (3/2/2/39/2020-NCD-III), Government of India, for fellowship supports.

## Conflict of Interest

The authors declare that the research was conducted in the absence of any commercial or financial relationships that could be construed as a potential conflict of interest.

## Publisher’s Note

All claims expressed in this article are solely those of the authors and do not necessarily represent those of their affiliated organizations, or those of the publisher, the editors and the reviewers. Any product that may be evaluated in this article, or claim that may be made by its manufacturer, is not guaranteed or endorsed by the publisher.

## References

[1] Sirajuddin M Rice LM Vale RD. Regulation of Microtubule Motors by Tubulin Isotypes and Post-Translational Modifications. Nat Cell Biol (2014) 16:335–44. doi: 10.1038/ncb2920 PMC4117587 24633327

[2] Gadadhar S Hirschmugl T Janke C. The Tubulin Code in Mammalian Sperm Development and Function. Semin Cell Dev Biol (2022). doi: 10.1016/j.semcdb.2021.12.003 35067438

[3] Vemu A Atherton J Spector JO Moores CA Roll-Mecak A. Tubulin Isoform Composition Tunes Microtubule Dynamics. Mol Biol Cell (2017) 28:3564–72. doi: 10.1091/mbc.E17-02-0124 PMC5706985 29021343

[4] Carre M Andre N Carles G Borghi H Brichese L Briand C. Tubulin Is an Inherent Component of Mitochondrial Membranes That Interacts With the Voltage-Dependent Anion Channel. J Biol Chem (2002) 277:33664–9. doi: 10.1074/jbc.M203834200 12087096

[5] Dharmapal D Jyothy A Mohan A Balagopal PG George NA Sebastian P. Beta-Tubulin Isotype, TUBB4B, Regulates The Maintenance of Cancer Stem Cells. Front Oncol (2021) 11:788024. doi: 10.3389/fonc.2021.788024 35004310 PMC8733585

[6] Guda MR Labak CM Omar SI Asuthkar S Airala S Tuszynski J. GLUT1 and TUBB4 in Glioblastoma Could be Efficacious Targets. Cancers (Basel) (2019) 11. doi: 10.3390/cancers11091308 PMC6771132 31491891

[7] Jurewicz E Wyroba E Filipek A. ubulin-Dependent Secretion of S100A6 and Cellular Signaling Pathways Activated by S100A6-Integrin Beta1 Interaction. Cell Signal (2018) 42:21–9. doi: 10.1016/j.cellsig.2017.10.004 29020611

[8] Kang EY Ponzio M Gupta PP Liu F Butensky A Gutstein DE. Identification of Binding Partners for the Cytoplasmic Loop of Connexin43: A Novel Interaction With Beta-Tubulin. Cell Commun Adhes (2009) 15:397–406. doi: 10.1080/15419060902783833 19274588 PMC2889002

[9] Chakraborty S Nagashri MN Mohiyuddin SM Gopinath KS Kumar A. Gene Expression Profiling of Oral Squamous Cell Carcinoma by Differential Display Rt-PCR and Identification of Tumor Biomarkers. Indian J Surg Oncol (2010) 1:284–93. doi: 10.1007/s13193-011-0054-x PMC3244262 22693380

[10] Parker AL Teo WS Mccarroll JA Kavallaris M. An Emerging Role for Tubulin Isotypes in Modulating Cancer Biology and Chemotherapy Resistance. Int J Mol Sci (2017) 18. doi: 10.3390/ijms18071434 PMC5535925 28677634

[11] Parker AL Kavallaris M Mccarroll JA. Microtubules and Their Role in Cellular Stress in Cancer. Front Oncol (2014) 4:153. doi: 10.3389/fonc.2014.00153 24995158 PMC4061531

[12] Vasudevan S Thomas SA Sivakumar KC Komalam RJ Sreerekha KV Rajasekharan KN. Diaminothiazoles Evade Multidrug Resistance in Cancer Cells and Xenograft Tumour Models and Develop Transient Specific Resistance: Understanding the Basis of Broad-Spectrum Versus Specific Resistance. Carcinogenesis (2015) 36:883–93. doi: 10.1093/carcin/bgv072 26014355

[13] Barr FA Egerer J. Golgi Positioning: Are We Looking at the Right MAP? J Cell Biol (2005) 168:993–8. doi: 10.1083/jcb.200501088 PMC2171853 15781478

[14] Lane JD Allan VJ. Microtubule-Based Endoplasmic Reticulum Motility in Xenopus Laevis: Activation of Membrane-Associated Kinesin During Development. Mol Biol Cell (1999) 10:1909–22. doi: 10.1091/mbc.10.6.1909 PMC25389 10359605

[15] Melkov A Abdu U. Regulation of Long-Distance Transport of Mitochondria Along Microtubules. Cell Mol Life Sci (2018) 75:163–76. doi: 10.1007/s00018-017-2590-1 PMC11105322 28702760

[16] Hanspal M Palek J. Synthesis and Assembly of Membrane Skeletal Proteins in Mammalian Red Cell Precursors. J Cell Biol (1987) 105:1417–24. doi: 10.1083/jcb.105.3.1417 PMC2114789 3654760

[17] Pu J Guardia CM Keren-Kaplan T Bonifacino JS. Mechanisms and Functions of Lysosome Positioning. J Cell Sci (2016) 129:4329–39. doi: 10.1242/jcs.196287 PMC5201012 27799357

[18] Bodakuntla S Jijumon AS Villablanca C Gonzalez-Billault C Janke C. Microtubule-Associated Proteins: Structuring the Cytoskeleton. Trends Cell Biol (2019) 29:804–19. doi: 10.1016/j.tcb.2019.07.004 31416684

[19] Naghavi MH Walsh D. Microtubule Regulation and Function During Virus Infection. J Virol (2017) 91. doi: 10.1128/JVI.00538-17 PMC5533906 28615197

[20] Barlan K Gelfand VI. Microtubule-Based Transport and the Distribution, Tethering, and Organization of Organelles. Cold Spring Harb Perspect Biol (2017) 9. doi: 10.1101/cshperspect.a025817 PMC5411697 28461574

[21] Akhmanova A Hammer JA 3rd. Linking Molecular Motors to Membrane Cargo. Curr Opin Cell Biol (2010) 22:479–87. doi: 10.1016/j.ceb.2010.04.008 PMC3393125 20466533

[22] Spiliotis ET Kesisova IA. Spatial Regulation of Microtubule-Dependent Transport by Septin GTPases. Trends Cell Biol (2021) 31:979–93. doi: 10.1016/j.tcb.2021.06.004 PMC8595587 34253430

[23] Verhey KJ Meyer D Deehan R Blenis J Schnapp BJ Rapoport TA. Cargo of Kinesin Identified as JIP Scaffolding Proteins and Associated Signaling Molecules. J Cell Biol (2001) 152:959–70. doi: 10.1083/jcb.152.5.959 PMC2198804 11238452

[24] Reed NA Cai D Blasius TL Jih GT Meyhofer E Gaertig J. Microtubule Acetylation Promotes Kinesin-1 Binding and Transport. Curr Biol (2006) 16:2166–72. doi: 10.1016/j.cub.2006.09.014 17084703

[25] Walter WJ Beranek V Fischermeier E Diez S. Tubulin Acetylation Alone Does Not Affect Kinesin-1 Velocity and Run Length *In Vitro* . PloS One (2012) 7:e42218. doi: 10.1371/journal.pone.0042218 22870307 PMC3411631

[26] Janke C Magiera MM. The Tubulin Code and its Role in Controlling Microtubule Properties and Functions. Nat Rev Mol Cell Biol (2020) 21:307–26. doi: 10.1038/s41580-020-0214-3 32107477

[27] Yildiz A. Sorting Out Microtubule-Based Transport. Nat Rev Mol Cell Biol (2021) 22:73. doi: 10.1038/s41580-020-00320-y 33288890

[28] Wawro ME Sobierajska K Ciszewski WM Wagner W Frontczak M Wieczorek K. Tubulin Beta 3 and 4 Are Involved in the Generation of Early Fibrotic Stages. Cell Signal (2017) 38:26–38. doi: 10.1016/j.cellsig.2017.06.014 28648944

[29] Im S Yoo C Jung JH Jeon YW Suh YJ Lee YS. Microtubule-Associated Protein Tau, Alpha-Tubulin and betaIII-Tubulin Expression in Breast Cancer. Korean J Pathol (2013) 47:534–40. doi: 10.4132/KoreanJPathol.2013.47.6.534 PMC3887155 24421846

[30] Boggs AE Vitolo MI Whipple RA Charpentier MS Goloubeva OG Ioffe OB. Alpha-Tubulin Acetylation Elevated in Metastatic and Basal-Like Breast Cancer Cells Promotes Microtentacle Formation, Adhesion, and Invasive Migration. Cancer Res (2015) 75:203–15. doi: 10.1158/0008-5472.CAN-13-3563 PMC4350791 25503560

[31] Nami B Wang Z. Genetics and Expression Profile of the Tubulin Gene Superfamily in Breast Cancer Subtypes and Its Relation to Taxane Resistance. Cancers (Basel) (2018) 10(8). doi: 10.10.3390/cancers10080274 PMC6116153 30126203

[32] Hasegawa S Miyoshi Y Egawa C Ishitobi M Taguchi T Tamaki Y. Prediction of Response to Docetaxel by Quantitative Analysis of Class I and III Beta-Tubulin Isotype mRNA Expression in Human Breast Cancers. Clin Cancer Res (2003) 9:2992–7. 12912947

[33] Fang W Li X Jiang Q Liu Z Yang H Wang S. Transcriptional Patterns, Biomarkers and Pathways Characterizing Nasopharyngeal Carcinoma of Southern China. J Transl Med (2008) 6:32. doi: 10.1186/1479-5876-6-32 18570662 PMC2443113

[34] Ruksha K Mezheyeuski A Nerovnya A Bich T Tur G Gorgun J. Over-Expression of betaII-Tubulin and Especially Its Localization in Cell Nuclei Correlates With Poorer Outcomes in Colorectal Cancer. Cells (2019) 8. doi: 10.3390/cells8010025 PMC6357106 30621030

[35] Yeh IT Luduena RF. The betaII Isotype of Tubulin Is Present in the Cell Nuclei of a Variety of Cancers. Cell Motil Cytoskeleton (2004) 57:96–106. doi: 10.1002/cm.10157 14691949

[36] Shin D Park J Han D Moon JH Ryu HS Kim Y. Identification of TUBB2A by Quantitative Proteomic Analysis as a Novel Biomarker for the Prediction of Distant Metastatic Breast Cancer. Clin Proteomics (2020) 17:16. doi: 10.1186/s12014-020-09280-z 32489334 PMC7247212

[37] Zheng W Yang C Qiu L Feng X Sun K Deng H. Transcriptional Information Underlying the Generation of CSCs and the Construction of a Nine-mRNA Signature to Improve Prognosis Prediction in Colorectal Cancer. Cancer Biol Ther (2020) 21:688–97. doi: 10.1080/15384047.2020.1762419 PMC7515529 32453965

[38] Chen L Xiang Z Chen X Zhu X Peng XA. Seven-Gene Signature Model Predicts Overall Survival in Kidney Renal Clear Cell Carcinoma. Hereditas (2020) 157:38. doi: 10.1186/s41065-020-00152-y 32883362 PMC7470605

[39] Sekino Y Han X Babasaki T Miyamoto S Kitano H Kobayashi G. TUBB3 Is Associated With High-Grade Histology, Poor Prognosis, P53 Expression, and Cancer Stem Cell Markers in Clear Cell Renal Cell Carcinoma. Oncology (2020) 98:689–98. doi: 10.1159/000506775 32585672

[40] Maahs L Sanchez BE Gupta N Van Harn M Barrack ER Reddy PV. Class III Beta-Tubulin Expression as a Predictor of Docetaxel-Resistance in Metastatic Castration-Resistant Prostate Cancer. PloS One (2019) 14:e0222510. doi: 10.1371/journal.pone.0222510 31658275 PMC6816559

[41] Oztop S Isik A Guner G Gurdal H Karabulut E Yilmaz E. Class III Beta-Tubulin Expression in Colorectal Neoplasms Is a Potential Predictive Biomarker for Paclitaxel Response. Anticancer Res (2019) 39:655–62. doi: 10.21873/anticanres.13160 30711942

[42] Nienstedt JC Grobe A Clauditz T Simon R Muenscher A Knecht R. High-Level betaIII-Tubulin Overexpression Occurs in Most Head and Neck Cancers But Is Unrelated to Clinical Outcome. J Oral Pathol Med (2017) 46:986–90. doi: 10.1111/jop.12607 28640948

[43] Christoph DC Kasper S Gauler TC Loesch C Engelhard M Theegarten D. betaV-Tubulin Expression Is Associated With Outcome Following Taxane-Based Chemotherapy in Non-Small Cell Lung Cancer. Br J Cancer (2012) 107:823–30. doi: 10.1038/bjc.2012.324 PMC3425975 22836512

[44] Kanakkanthara A Miller JH. betaIII-Tubulin Overexpression in Cancer: Causes, Consequences, and Potential Therapies. Biochim Biophys Acta Rev Cancer (2021) 1876:188607. doi: 10.1016/j.bbcan.2021.188607 34364992

[45] Lopes D Maiato H. The Tubulin Code in Mitosis and Cancer. Cells (2020) 9. doi: 10.3390/cells9112356 PMC7692294 33114575

[46] Prassanawar SS Panda D. Tubulin Heterogeneity Regulates Functions and Dynamics of Microtubules and Plays a Role in the Development of Drug Resistance in Cancer. Biochem J (2019) 476:1359–76. doi: 10.1042/BCJ20190123 31085712

[47] Wang Z Chen J Wang J Ahn S Li CM Lu Y. Novel Tubulin Polymerization Inhibitors Overcome Multidrug Resistance and Reduce Melanoma Lung Metastasis. Pharm Res (2012) 29:3040–52. doi: 10.1007/s11095-012-0726-4 PMC3659804 22410804

[48] Wattanathamsan O Thararattanobon R Rodsiri R Chanvorachote P Vinayanuwattikun C Pongrakhananon V. Tubulin Acetylation Enhances Lung Cancer Resistance to Paclitaxel-Induced Cell Death Through Mcl-1 Stabilization. Cell Death Discov (2021) 7:67. doi: 10.1038/s41420-021-00453-9 33824297 PMC8024319

[49] Wattanathamsan O Pongrakhananon V. Post-Translational Modifications of Tubulin: Their Role in Cancers and the Regulation of Signaling Molecules. Cancer Gene Ther (2021). doi: 10.1038/s41417-021-00396-4 34671113

[50] Downing KH. Structural Basis for the Interaction of Tubulin With Proteins and Drugs That Affect Microtubule Dynamics. Annu Rev Cell Dev Biol (2000) 16:89–111. doi: 10.1146/annurev.cellbio.16.1.89 11031231

[51] Sobierajska K Ciszewski WM Wawro ME Wieczorek-Szukala K Boncela J Papiewska-Pajak I. TUBB4B Downregulation Is Critical for Increasing Migration of Metastatic Colon Cancer Cells. Cells (2019) 8. doi: 10.3390/cells8080810 PMC6721557 31375012

[52] Wang Q Liu X. VDAC Upregulation and Alphatat1mediated Alphatubulin Acetylation Contribute to Tanespimycininduced Apoptosis in Calu1 Cells. Oncol Rep (2020) 44:2725–34. doi: 10.3892/or.2020.7789 33125132

[53] Kunz TH Mueller-Steiner S Schwerdtfeger K Kleinert P Troxler H Kelm JM. Interaction of Receptor-Activity-Modifying Protein1 With Tubulin. Biochim Biophys Acta (2007) 1770:1145–50. doi: 10.1016/j.bbagen.2007.04.002 17493758

[54] Bracey K Ju M Tian C Stevens L Wray D. Tubulin as a Binding Partner of the Heag2 Voltage-Gated Potassium Channel. J Membr Biol (2008) 222:115–25. doi: 10.1007/s00232-008-9104-x 18458804

[55] Yeh TS Hsieh RH Shen SC Wang SH Tseng MJ Shih CM. Nuclear betaII-Tubulin Associates With the Activated Notch Receptor to Modulate Notch Signaling. Cancer Res (2004) 64:8334–40. doi: 10.1158/0008-5472.CAN-04-2197 15548702

[56] Cicchillitti L Penci R Di Michele M Filippetti F Rotilio D Donati MB. Proteomic Characterization of Cytoskeletal and Mitochondrial Class III Beta-Tubulin. Mol Cancer Ther (2008) 7:2070–9. doi: 10.1158/1535-7163.MCT-07-2370 18645017

[57] Puurand M Tepp K Timohhina N Aid J Shevchuk I Chekulayev V. Tubulin betaII and betaIII Isoforms as the Regulators of VDAC Channel Permeability in Health and Disease. Cells (2019) 8. doi: 10.3390/cells8030239 PMC6468622 30871176

[58] Kumar B Kumar R Skvortsova I Kumar V. Mechanisms of Tubulin Binding Ligands to Target Cancer Cells: Updates on Their Therapeutic Potential and Clinical Trials. Curr Cancer Drug Targets (2017) 17:357–75. doi: 10.2174/1568009616666160928110818 27697026

[59] Huang X Dubuc AM Hashizume R Berg J He Y Wang J. Voltage-Gated Potassium Channel EAG2 Controls Mitotic Entry and Tumor Growth in Medulloblastoma *via* Regulating Cell Volume Dynamics. Genes Dev (2012) 26:1780–96. doi: 10.1101/gad.193789.112 PMC3426758 22855790

[60] Ai XL Chi Q Qiu Y Li HY Li DJ Wang JX. Gap Junction Protein Connexin43 Deregulation Contributes to Bladder Carcinogenesis via Targeting MAPK Pathway. Mol Cell Biochem (2017) 428:109–18. doi: 10.1007/s11010-016-2921-9 28074341

[61] Han Y Zhang PJ Chen T Yum SW Pasha T Furth EE. Connexin43 Expression Increases in the Epithelium and Stroma Along the Colonic Neoplastic Progression Pathway: Implications for Its Oncogenic Role. Gastroenterol Res Pract (2011) 2011:561719. doi: 10.1155/2011/561719 21754925 PMC3132986

[62] Kazan JM El-Saghir J Saliba J Shaito A Jalaleddine N El-Hajjar L. Cx43 Expression Correlates With Breast Cancer Metastasis in MDA-MB-231 Cells *In Vitro*, In a Mouse Xenograft Model and in Human Breast Cancer Tissues. Cancers (Basel) (2019) 11. doi: 10.3390/cancers11040460 PMC6521103 30939738

[63] Lamiche C Clarhaut J Strale PO Crespin S Pedretti N Bernard FX. The Gap Junction Protein Cx43 Is Involved in the Bone-Targeted Metastatic Behaviour of Human Prostate Cancer Cells. Clin Exp Metastasis (2012) 29:111–22. doi: 10.1007/s10585-011-9434-4 22080401

[64] Deng S Krutilina RI Wang Q Lin Z Parke DN Playa HC. An Orally Available Tubulin Inhibitor, VERU-111, Suppresses Triple-Negative Breast Cancer Tumor Growth and Metastasis and Bypasses Taxane Resistance. Mol Cancer Ther (2020) 19:348–63. doi: 10.1158/1535-7163.MCT-19-0536 PMC7007836 31645441

[65] Liu H Fu Q Lu Y Zhang W Yu P Liu Z. Anti-Tubulin Agent Vinorelbine Inhibits Metastasis of Cancer Cells by Regulating Epithelial-Mesenchymal Transition. Eur J Med Chem (2020) 200:112332. doi: 10.1016/j.ejmech.2020.112332 32473523

[66] Abbassi RH Recasens A Indurthi DC Johns TG Stringer BW Day BW. Lower Tubulin Expression in Glioblastoma Stem Cells Attenuates Efficacy of Microtubule-Targeting Agents. ACS Pharmacol Transl Sci (2019) 2:402–13. doi: 10.1021/acsptsci.9b00045 PMC7089104 32259073

[67] Bao L Xu T Lu X Huang P Pan Z Ge M. Metabolic Reprogramming of Thyroid Cancer Cells and Crosstalk in Their Microenvironment. Front Oncol (2021) 11:773028. doi: 10.3389/fonc.2021.773028 34926283 PMC8674491

[68] Kareva I Hahnfeldt P. The Emerging “Hallmarks” of Metabolic Reprogramming and Immune Evasion: Distinct or Linked? Cancer Res (2013) 73:2737–42. doi: 10.1158/0008-5472.CAN-12-3696 23423980

[69] Shen YA Chen CC Chen BJ Wu YT Juan JR Chen LY. Potential Therapies Targeting Metabolic Pathways in Cancer Stem Cells. Cells (2021) 10(7):1772. doi: 10.10.3390/cells10071772 34359941 PMC8304173

[70] Aguilar E Marin De Mas I Zodda E Marin S Morrish F Selivanov V. Metabolic Reprogramming and Dependencies Associated With Epithelial Cancer Stem Cells Independent of the Epithelial-Mesenchymal Transition Program. Stem Cells (2016) 34:1163–76. doi: 10.1002/stem.2286 PMC4860823 27146024

[71] Menendez JA Joven J Cufi S Corominas-Faja B Oliveras-Ferraros C Cuyas E. The Warburg Effect Version 2.0: Metabolic Reprogramming of Cancer Stem Cells. Cell Cycle (2013) 12:1166–79. doi: 10.4161/cc.24479 PMC3674082 23549172

[72] Maldonado EN. VDAC-Tubulin, an Anti-Warburg Pro-Oxidant Switch. Front Oncol (2017) 7:4. doi: 10.3389/fonc.2017.00004 28168164 PMC5256068

[73] Kim J Cheong JH. Role of Mitochondria-Cytoskeleton Interactions in the Regulation of Mitochondrial Structure and Function in Cancer Stem Cells. Cells (2020) 9. doi: 10.3390/cells9071691 PMC7407978 32674438

[74] Gadau S Lepore G Zedda M Manca P Chisu V Farina V. D-Glucose Induces Microtubular Changes in C1300 Neuroblastoma Cell Line Through the Incorporation of 3-Nitro-L-Tyrosine Into Tubulin. Arch Ital Biol (2008) 146:107–17. 18822798

[75] Konishi Y Setou M. Tubulin Tyrosination Navigates the Kinesin-1 Motor Domain to Axons. Nat Neurosci (2009) 12:559–67. doi: 10.1038/nn.2314 19377471

[76] Nirschl JJ Magiera MM Lazarus JE Janke C Holzbaur EL. Alpha-Tubulin Tyrosination and CLIP-170 Phosphorylation Regulate the Initiation of Dynein-Driven Transport in Neurons. Cell Rep (2016) 14:2637–52. doi: 10.1016/j.celrep.2016.02.046 PMC4819336 26972003

[77] Hamann I Krys D Glubrecht D Bouvet V Marshall A Vos L. Expression and Function of Hexose Transporters GLUT1, GLUT2, and GLUT5 in Breast Cancer-Effects of Hypoxia. FASEB J (2018) 32:5104–18. doi: 10.1096/fj.201800360R 29913554

[78] Ouiddir A Planes C Fernandes I Vanhesse A Clerici C. Hypoxia Upregulates Activity and Expression of the Glucose Transporter GLUT1 in Alveolar Epithelial Cells. Am J Respir Cell Mol Biol (1999) 21:710–8. doi: 10.1165/ajrcmb.21.6.3751 10572068

[79] Chen XH Liu J Zhong JT Zhou SH Fan J. Effect of GLUT1 Inhibition and Autophagy Modulation on the Growth and Migration of Laryngeal Carcinoma Stem Cells Under Hypoxic and Low-Glucose Conditions. Onco Targets Ther (2021) 14:3069–81. doi: 10.2147/OTT.S300423 PMC8124017 34007184

[80] Kim H Ju JH Son S Shin I. Silencing of CD133 Inhibits GLUT1-Mediated Glucose Transport Through Downregulation of the HER3/Akt/mTOR Pathway in Colon Cancer. FEBS Lett (2020) 594:1021–35. doi: 10.1002/1873-3468.13686 31736063

[81] Shibuya K Okada M Suzuki S Seino M Seino S Takeda H. Targeting the Facilitative Glucose Transporter GLUT1 Inhibits the Self-Renewal and Tumor-Initiating Capacity of Cancer Stem Cells. Oncotarget (2015) 6:651–61. doi: 10.18632/oncotarget.2892 PMC4359246 25528771

[82] Parker AL Turner N Mccarroll JA Kavallaris M. betaIII-Tubulin Alters Glucose Metabolism and Stress Response Signaling to Promote Cell Survival and Proliferation in Glucose-Starved Non-Small Cell Lung Cancer Cells. Carcinogenesis (2016) 37:787–98. doi: 10.1093/carcin/bgw058 27207668

[83] Li Z Wang Y Wu H Zhang L Yang P Li Z. GRP78 Enhances the Glutamine Metabolism to Support Cell Survival From Glucose Deficiency by Modulating the Beta-Catenin Signaling. Oncotarget (2014) 5:5369–80. doi: 10.18632/oncotarget.2105 PMC4170599 24977433

[84] Gopal U Pizzo SV. Cell Surface GRP78 Promotes Tumor Cell Histone Acetylation Through Metabolic Reprogramming: A Mechanism Which Modulates the Warburg Effect. Oncotarget (2017) 8:107947–63. doi: 10.18632/oncotarget.22431 PMC5746117 29296215

[85] Gopal U Pizzo SV. Cell Surface GRP78 Signaling: An Emerging Role as a Transcriptional Modulator in Cancer. J Cell Physiol (2021) 236:2352–63. doi: 10.1002/jcp.30030 32864780

[86] Alvarez-Trotta A Guerrant W Astudillo L Lahiry M Diluvio G Shersher E. Pharmacological Disruption of the Notch1 Transcriptional Complex Inhibits Tumor Growth by Selectively Targeting Cancer Stem Cells. Cancer Res (2021) 81:3347–57. doi: 10.1158/0008-5472.CAN-20-3611 PMC8655881 33820800

[87] Cai H Lu W Zhang Y Liu H Wang Z Shen Y. Specific Inhibition of Notch1 Signaling Suppresses Properties of Lung Cancer Stem Cells. J Cancer Res Ther (2019) 15:1547–52. doi: 10.4103/jcrt.JCRT_482_17 31939436

[88] Du Y Shao H Moller M Prokupets R Tse YT Liu ZJ. Intracellular Notch1 Signaling in Cancer-Associated Fibroblasts Dictates the Plasticity and Stemness of Melanoma Stem/Initiating Cells. Stem Cells (2019) 37:865–75. doi: 10.1002/stem.3013 PMC6986496 30941836

[89] Zhang Y Xu W Guo H Zhang Y He Y Lee SH. NOTCH1 Signaling Regulates Self-Renewal and Platinum Chemoresistance of Cancer Stem-Like Cells in Human Non-Small Cell Lung Cancer. Cancer Res (2017) 77:3082–91. doi: 10.1158/0008-5472.CAN-16-1633 28416482

[90] Koo BK Lim HS Chang HJ Yoon MJ Choi Y Kong MP. Notch Signaling Promotes the Generation of EphrinB1-Positive Intestinal Epithelial Cells. Gastroenterology (2009) 137:145–55 55.0e1–3. doi: 10.1053/j.gastro.2009.03.046 19332065

[91] Yu L Xia K Gao T Chen J Zhang Z Sun X. The Notch Pathway Promotes Osteosarcoma Progression Through Activation of Ephrin Reverse Signaling. Mol Cancer Res (2019) 17:2383–94. doi: 10.1158/1541-7786.MCR-19-0493 31570655

[92] Tsuchiya H Shiota G. Immune Evasion by Cancer Stem Cells. Regener Ther (2021) 17:20–33. doi: 10.1016/j.reth.2021.02.006 PMC7966825 33778133

[93] Lei MML Lee TKW. Cancer Stem Cells: Emerging Key Players in Immune Evasion of Cancers. Front Cell Dev Biol (2021) 9:692940. doi: 10.3389/fcell.2021.692940 34235155 PMC8257022

[94] Fan C Zhang S Gong Z Li X Xiang B Deng H. Emerging Role of Metabolic Reprogramming in Tumor Immune Evasion and Immunotherapy. Sci China Life Sci (2021) 64:534–47. doi: 10.1007/s11427-019-1735-4 32815067

[95] Luan JC Zhang QJ Zhao K Zhou X Yao LY Zhang TT. A Novel Set of Immune-Associated Gene Signature Predicts Biochemical Recurrence in Localized Prostate Cancer Patients After Radical Prostatectomy. J Cancer (2021) 12:3715–25. doi: 10.7150/jca.51059 PMC8120173 33995646

[96] Martin-Cofreces NB Sanchez-Madrid F. Sailing to and Docking at the Immune Synapse: Role of Tubulin Dynamics and Molecular Motors. Front Immunol (2018) 9:1174. doi: 10.3389/fimmu.2018.01174 29910809 PMC5992405

[97] Fong A Durkin A Lee H. The Potential of Combining Tubulin-Targeting Anticancer Therapeutics and Immune Therapy. Int J Mol Sci (2019) 20. doi: 10.3390/ijms20030586 PMC6387102 30704031

[98] Uchida Y Matsushima T Kurimoto R Chiba T Inutani Y Asahara H. Identification of Chemical Compounds Regulating PD-L1 by Introducing HiBiT-Tagged Cells. FEBS Lett (2021) 595:563–76. doi: 10.1002/1873-3468.14032 PMC7940577 33421110

[99] Shen LF Zhou SH Guo Y. Role of GLUT-1 in the Upregulation of PD-L1 Expression After Radiotherapy and Association of PD-L1 With Favourable Overall Survival in Hypopharyngeal Cancer. Onco Targets Ther (2020) 13:11221–35. doi: 10.2147/OTT.S269767 PMC7648563 33173312

[100] Chou CW Yang RY Chan LC Li CF Sun L Lee HH. The Stabilization of PD-L1 by the Endoplasmic Reticulum Stress Protein GRP78 in Triple-Negative Breast Cancer. Am J Cancer Res (2020) 10:2621–34. PMC7471351 32905506

[101] Yang WH Cha JH Xia W Lee HH Chan LC Wang YN. Juxtacrine Signaling Inhibits Antitumor Immunity by Upregulating PD-L1 Expression. Cancer Res (2018) 78:3761–68. doi: 10.1158/0008-5472.CAN-18-0040 PMC6050079 29789418

